# Analysis of clinical research coordinator (CRC) deviation from assigned responsibilities in Sichuan, China based on empirical investigation

**DOI:** 10.3389/fmed.2026.1744512

**Published:** 2026-02-03

**Authors:** Shuxia Shi, Shangyuan Qin, Qin Yu

**Affiliations:** 1 National Drug Clinical Trial Institution of West China Second University Hospital, Sichuan University, Chengdu, China; 2 NMPA Key Laboratory for Technical Research on Drug Products In Vitro and In Vivo Correlation, Chengdu, China; 3 Children’s Medicine Key Laboratory of Sichuan Province, Chengdu, China; 4 Key Laboratory of Birth Defects and Related Diseases of Women and Children, Sichuan University, Ministry of Education, Chengdu, China; 5 Department of Pharmacy, Chengdu Fifth People’s Hospital, Chengdu, China

**Keywords:** clinical research coordinator, deviation from assigned responsibilities, questionnaire survey, responsibility, subject safety

## Abstract

**Objective:**

Exploring the current status and causes of overstepping behavior among clinical research coordinators: providing reference for standardizing CRC responsibilities and safeguarding clinical trial quality and subject safety.

**Methods:**

The research utilized a literature review and semi-structured interviews to design the survey questionnaire, which was disseminated online through WeChat via a Questionnaire Star link. Data were processed for missing values and outliers using IBM SPSS Statistics 29.0 and R software program (version 4.1.2), with categorical variables summarized using frequencies and percentages. The questionnaire was designed independently by our research team (excluding members from participating institutions) and pre-tested on several non-participating clinical trial professionals to verify neutrality. Semi-structured interviews were conducted by two researchers unaffiliated with participating institutions, with cross-checks for consistency.

**Results:**

The self-designed questionnaire in this study comprised 15 closed-ended questions (eight single-choice and seven multiple-choice items) and two open-ended questions. Both the Scale-level Content Validity Index/Universal Agreement (S-CVI/UA) and the Scale-level Content Validity Index/Average (S-CVI/Ave) exceeded 0.9, while Cronbach’s Alpha coefficient was greater than 0.7, indicating high overall content validity and reliability of the questionnaire. A total of 202 stakeholders in clinical trials participated in the survey, including staff from clinical trial institutions, sponsors/CROs, and SMO personnel. Survey results indicated that 177 respondents (33.71%/87.62%) attributed CRC role overstepping to investigators’ unclear understanding of their own responsibilities and those of CRCs. Meanwhile, 181 participants (38.11%/89.60%) reported that such overstepping frequently occurred when investigators requested CRCs to perform potentially unauthorized tasks. The most common type of overstepping, identified by 152 respondents (31.93%/75.25%), involved CRCs using physicians’ accounts to access hospital management systems for medical record documentation or modification. One hundred seventy-seven participants (29.70%/87.62%) proposed that strengthening investigator management, specifically by prohibiting the sharing of personal login credentials, should be implemented as a key control measure. Chi-square test performed on the collected data revealed no statistically significant differences (*p* > 0.05) in pairwise comparisons between subgroups.

**Conclusion:**

CRC role boundary violations frequently correlate with investigators’ insufficient awareness of regulatory requirements and clinical trial protocols. As principal contributors to clinical research, investigators must clearly delineate and fulfill their essential trial-related obligations. Concurrently, CRCs should strictly adhere to Good Clinical Practice (GCP) guidelines and industry standards while assisting investigators, thereby collectively enhancing trial efficiency and quality.

## 1 Introduction

With the widespread development of drug and medical device clinical trials in various medical institutions both domestically and internationally, a new professional group has emerged within this field: the Clinical Research Coordinator (CRC). The global demand for CRCs continues to expand. For instance, the U.S. employment market for CRCs is projected to grow by 9.9% between 2016 and 2026 (1, 2). In China, the CRC workforce has expanded at an annual rate of 15% since 2020, with the total number exceeding 120,000 by 2024 (3), outpacing the U.S. growth rate and reflecting the rapid development of clinical trials in China. While their increasing presence creates opportunities, it also presents significant challenges. Since July 22, 2015, China’s drug regulatory authorities have implemented the “strictest” clinical trial data verification standards, making it particularly challenging to meet audit requirements without CRC involvement (4, 5). The strengthening of clinical trial regulations has elevated expectations for CRCs, necessitating greater emphasis on skills, training, and medical knowledge (6). A stable and experienced CRC workforce has become crucial for ensuring clinical trial quality. A CRC is an individual possessing requisite educational qualifications who has undergone relevant training and is authorized by the Principal Investigator (PI). Functioning as a member of the research team, the CRC undertakes tasks related to the clinical trial that do not involve medical judgment. The CRC can serve as an implementer, coordinator, and administrator within the clinical trial (7, 8). CRCs are site-based professionals authorized to assist with non-medical trial tasks (e.g., data entry, subject coordination), while Clinical Research Associates (CRAs) are sponsor/CRO-affiliated monitors responsible for cross-site trial compliance. This study focuses exclusively on CRCs.

The earliest emergence of this novel profession occurred in Europe and the United States(1970), where nurses predominantly filled this role (9). Following the promulgation of ICH-GCP in 1996, Japan formally introduced the concept of CRCs during its April 1997 revision of Good Clinical Practice (GCP). The Japanese guidelines explicitly defined CRCs as a component of the clinical trial support system, distinguishing their role from that of CRAs. At this stage, most CRCs in Japan possessed a pharmaceutical academic background. In contrast, the emergence of CRCs in China did not occur until the late 1990s. Initially, the role was predominantly filled by part-time clinical physicians or medical postgraduate students, reflecting a different developmental trajectory compared to Japan. Subsequently, individuals from diverse professional backgrounds, including nurses, pharmacists, laboratory technicians, and institutional management personnel, gradually transitioned into CRC roles (3, 5).

In recent years, current laws and regulations issued by China’s clinical trial regulatory authorities have clearly delineated the responsibilities of investigators, sponsors, and CRAs. However, these policies fail to address or define the specific duties of CRCs. To address this gap, professional associations have actively engaged in developing industry standards and certification systems for CRC professionals. For instance: The Guangdong Clinical Trial Committee proposed the “Guangdong Consensus on CRC Management” (10); The Drug Clinical Trial Institution Alliance issued “Field-Specific Guidelines for Clinical Research Coordination” (11). Sponsor-related time pressure and resource constraints are emerging risk factors for CRC role deviation, as investigators may delegate unauthorized tasks to CRCs to meet trial timelines. This challenge is increasingly recognized in Chinese clinical trial practice (12).

Since 2020, the CRC profession has developed into an established industry. While consensus has been largely achieved regarding the responsibilities and tasks of CRCs, China currently lacks a comprehensive professionalization framework for this role (12). This systemic gap has resulted in ambiguous role delineation, frequently leading to role boundary violations in daily practice. Consequently, there is an urgent need to analyze the root causes of such violations and develop effective countermeasures. This study employs a questionnaire-based methodology to collect multi-stakeholder perceptions of CRC role boundary violations and their proposed control measures. Furthermore, it examines the direct risks arising from these violations and their implications for clinical trial quality.

## 2 Materials and methods

### 2.1 Study participants

From March to June 2025, we conducted an electronic questionnaire survey among personnel affiliated with drug clinical trial institutions in Sichuan Province that possess official clinical trial qualifications. This study was conducted exclusively among clinical trial practitioners in Sichuan Province, China, which may limit the generalizability of results to other regions. The target population comprised: Clinical trial professionals, Sponsor representatives, Contract Research Organization (CRO), Site Management Organization (SMO).

### 2.2 Data collection methods and survey instrument

The study employed a mixed-methods approach combining literature review, document analysis of regulatory findings, and semi-structured interviews to develop the Clinical Trial Site CRC Management Questionnaire. Guided by a systematic review of global literature (6, 7, 13, 14), we constructed a semi-structured interview framework derived from our initial survey instrument. The study encompasses the following key aspects: institutional categorization of survey respondents, the volume and temporal scope of clinical trials conducted by healthcare organizations, the professional duties of Clinical Research Coordinators (CRCs), the prevalence and underlying factors of CRC role boundary violations, along with their immediate risks and mitigation strategies. Refine and integrate the Clinical Trial Institution CRC Management Survey Questionnaire by synthesizing insights from interview results.

The survey was administered electronically using Questionnaire Star,^1^ a secure online data collection platform. An anonymous survey link was disseminated through WeChat, allowing self-administered responses. Datasets retrieved from the platform underwent systematic analysis and consolidation. The questionnaire was designed independently by our research team (excluding members from participating institutions) and pre-tested on several non-participating clinical trial professionals to verify neutrality. Semi-structured interviews were conducted by two researchers unaffiliated with participating institutions, with cross-checks for consistency.

### 2.3 Data analysis methods

#### 2.3.1 Statistical methods

Data were processed for missing values and outliers using IBM SPSS Statistics 29.0 and R software. The Complete Case Analysis (CCA) is was adopted for missing value processing in this study. Given that the sample size of responses to the open-ended questions in this study is small (fewer than 300), the method of “manual coding and categorization + frequency statistics + typical case extraction” was adopted. Single-choice questions were statistically described using frequencies and percentages. Multiple-choice questions were analyzed using multiple response analysis, including: Response rate (sum = 100%), which compares the relative selection frequency of each option. Penetration rate (sum > 100%), which indicates the prevalence of a particular choice among respondents (15). Group comparisons were analyzed by *χ*^2^ test or Fisher’s exact test (Fisher–Freeman–Halton test) (two-tailed; *α* = 0.05), with *p* < 0.05 defining statistical significance.

#### 2.3.2 Questionnaire reliability and validity assessment

The evaluation working group consisted of an experienced team from the institutional clinical trial center of our hospital. The content validity of the questionnaire (specifically for items 6–15, as the remaining items pertained to basic demographic information and thus did not require validity assessment) was evaluated using the Content Validity Index (CVI). This involved calculating the item-level CVI (I-CVI) as well as the scale-level CVI (S-CVI). The S-CVI was further categorized into: Scale-level CVI with universal agreement (S-CVI/UA); Average scale-level CVI (S-CVI/Ave). Through multiple rounds of evaluation and revision by the working group, the final Clinical Trial Institution CRC Management Survey Questionnaire was developed with high content validity. Additionally, the working group assessed the internal consistency reliability of the questionnaire using Cronbach’s alpha coefficient. A Cronbach’s alpha value greater than 0.7 for all scales was considered indicative of high reliability (16, 17).

## 3 Results

### 3.1 Questionnaire validity and reliability evaluation results

#### 3.1.1 Content validity assessment results

Five experts participated in evaluating the content validity of the Clinical Trial Institution CRC Management Survey Questionnaire. Item-level Content Validity Index (I-CVI): Among the 57 items (questions 6–15), 18 items had an I-CVI of 0.80, while the remaining items achieved an I-CVI of 1.00. All 10 major domains demonstrated excellent content validity (I-CVI = 1.00). Scale-level Content Validity Index (S-CVI): The universal agreement score (S-CVI/UA) was 0.91. The average agreement score (S-CVI/Ave) was 0.94. Both values exceeded the recommended threshold of 0.90, indicating strong overall content validity. These results confirm that the Clinical Trial Institution CRC Management Survey Questionnaire exhibits excellent content validity at both the item and scale levels.

#### 3.1.2 Questionnaire reliability assessment results

The internal consistency reliability of the Clinical Trial Institution CRC Management Survey Questionnaire was evaluated using Cronbach’s alpha coefficient. The analysis yielded a Cronbach’s alpha value of 0.91, which significantly exceeds the conventional threshold of 0.7. This result demonstrates excellent internal consistency among the assessment items, indicating high reliability of the questionnaire.

Item-level Content Validity Index (I-CVI): Among the 57 items (questions 6–15), 18 items had an I-CVI of 0.80, while the remaining items achieved an I-CVI of 1.00. All 10 major domains demonstrated excellent content validity (I-CVI = 1.00). Scale-level Content Validity Index (S-CVI): The universal agreement score (S-CVI/UA) was 0.91, the average agreement score (S-CVI/Ave) was 0.94. Cronbach’s alpha coefficient was 0.91, significantly exceeding the conventional threshold of 0.7.

### 3.2 Demographic characteristics of survey participants

This study employed a structured electronic questionnaire administered via the Questionnaire Star platform. A total of 202 clinical trial practitioners participated in this survey, among whom 135 were researchers from clinical trial institutions, accounting for 66.83% of the respondents. One participant was the director/president of the institution, and 15 were researchers from medical institutions (accounting for 7.43%). Clinical trial management personnel formed the largest subgroup (*n* = 71, 35.15%). The medical institutions that have undertaken GCP clinical trial projects for 1 to 5 years account for the largest proportion (43 institutions, accounting for 31.85%, with 135 valid participants). There are 26 institutions that have undertaken projects for more than 20 years (accounting for 19.26%, with 135 valid filling person-times). The institutions that undertake less than 5 clinical trials on average each year account for the largest proportion (48 institutions, accounting for 23.76%). The results of the above survey show that most of the medical institutions participating in this research are newly established. The relevant composition is shown in Table 1.

**Table 1:** Basic information of the respondents.

Group	Variable	Number (*N*)	Percent (%)
Source of study participants (Valid responses received: 202 participants)	Sponsor / CRO	36	17.82
	Clinical Trial Organization	135	66.83
	SMO	31	15.35
Respondent’s professional position (Valid responses received: 202 participants)	Institutional Director/President	1	0.5
	Director of Clinical Research Operations	71	35.15
	Clinical research operations personnel	43	21.29
	CRA employed by a Sponsor / CRO	21	10.4
	CRC employed by a SMO	30	14.85
	Director of Sponsor / CRO	7	3.47
	Director of SMO	2	0.99
	Healthcare facility-hired CRCs	8	3.96
	Investigator(s) at healthcare institutions	15	7.43
	Other	4	1.98
Average annual volume of GCP-compliant clinical trials conducted (Valid responses received: 202 participants)	≤5	48	23.76
	6–15	30	14.85
	16–30	33	16.34
	31–50	26	12.87
	51–100	36	17.82
	101–150	13	6.44
	151–200	9	4.46
	201–300	4	1.98
	>300	3	1.49
Years of experience in hosting GCP clinical trials (Valid responses received: 135 participants)	≤1 year	6	4.44
	1–5 year	43	31.85
	6–10 year	30	22.22
	10–20 year	30	22.22
	>20 year	26	19.26

### 3.3 Empirical analysis results

#### 3.3.1 CRC role perception

##### 3.3.1.1 Regulatory and guideline provisions on CRC responsibilities

1. Historical development of CRC in Foreign countries

The role of CRCs emerged in the United States as early as the 1970s, with their participation in clinical trials steadily increasing over time. Given the well-established history of CRCs in the U.S., academic institutions and professional associations have developed systematic training programs and certification standards for CRCs. The Association of Clinical Research Professionals (ACRP), founded in 1976, is a globally recognized non-profit organization that provides professional training and authoritative certification in clinical research. In 2021, ACRP published the industry’s first CRC Core Competency Guidelines, establishing standardized competency frameworks for CRC practice (18, 19).

1. Domestic regulatory references

In China, CRC practice is guided by the following key documents: Expert Consensus on Clinical Research Coordinator Practice and Management (2024 Edition) (6); Guangdong Consensus on CRC Management in Drug Clinical Trials (2024 Edition) (7); Industry Guidelines for Clinical Research Coordinators (CRC) (Trial Implementation) (20). In China, CRC practice is strictly governed by the Good Clinical Practice (GCP) for Drug Clinical Trials (21), which explicitly delineates CRCs’ permitted and prohibited activities, aligning with international ICH-GCP standards.

1. General Responsibilities

In clinical trial research, medical-related work such as informed notification, medical judgment, medical treatment, and disease course recording should be carried out by the research doctor and should not be authorized to the CRC.

##### 3.3.1.2 CRC daily responsibilities

**Figure 1:**
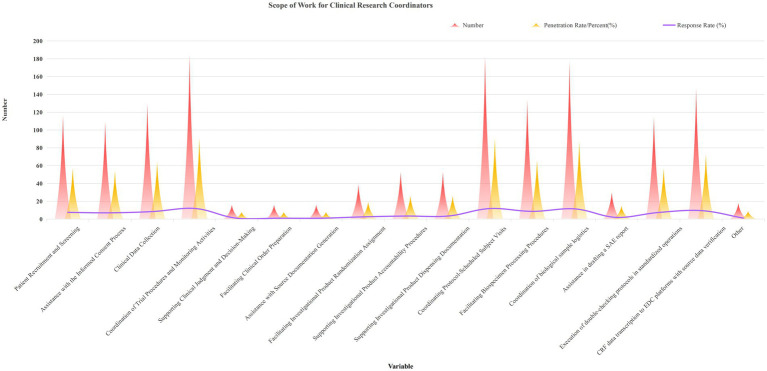
Scope of work for clinical research coordinators.

The survey results regarding CRC role responsibilities are presented in Table 2 and Figure 1. According to respondents’ perceptions, CRC daily duties primarily included (response rate/penetration rate): Trial process coordination & monitoring liaison (11.99%/91.09%); Follow-up visit scheduling (11.86%/90.10%); Specimen shipment coordination (11.53%/87.62%); EDC data entry (9.58%/72.77%); Specimen processing assistance (8.73%/66.34%); Data collection (8.49%/64.36%); Patient recruitment & screening (7.56%/57.43%); Operational verification (7.49%/56.93%); Informed consent process support (7.10%/53.96%). Among the 186 (92.08%) medical institutions participating in the survey, the working authority of CRC was clearly defined. Among them, 117 (57.92%) institutions had clear regulations in written documents and conducted training, learning and assessment. 30 (14.85%) institutions only conducted oral communication, and only 7.92% of medical institutions did not see any documents or Sops regarding the job responsibilities of CRCs. The results of the above survey content show that the medical institutions participating in this survey have a relatively high awareness of the job responsibilities of CRCs.

**Table 2:** Definition of CRC responsibilities and authorized scope of work.

Group	Variable	*N*	Response rate (RR) (%)	Penetration rate/percent (%)
Scope of work for clinical research coordinators (Valid responses received: 202 participants)	Patient recruitment and screening	116	7.56	57.43
	Assistance with the informed consent process	109	7.10	53.96
	Clinical data collection	130	8.49	64.36
	Coordination of trial procedures and monitoring activities	184	11.99	91.09
	Supporting clinical judgment and decision-making	16	1.04	7.92
	Facilitating clinical order preparation	16	1.04	7.92
	Assistance with source documentation generation	16	1.04	7.92
	Facilitating investigational product randomization assignment	39	2.54	19.31
	Supporting Investigational Product Accountability Procedures	53	3.45	26.24
	Supporting investigational product dispensing documentation	53	3.45	26.24
	Coordinating protocol-scheduled subject visits	182	11.86	90.1
	Facilitating biospecimen processing procedures	134	8.73	66.34
	Coordination of biological sample logistics	177	11.53	87.62
	Assistance in drafting a SAE report	30	1.95	14.85
	Execution of double-checking protocols in standardized operations	115	7.49	56.93
	CRF data transcription to EDC platforms with source data verification	147	9.58	72.77
	Other	18	1.17	8.91
Total		1,535	100.00%	759.91%
Delimitation of clinical research coordinator’s authorized activities (Valid responses received: 202 participants)	Yes, formal written procedures have been established, accompanied by mandatory training programs and competency verification measures.	117	NA	57.92
	Yes, verbal communication alone, without supporting written records.	30	NA	14.85
	Yes, explicitly stipulated in institutional documents yet lacking systematic training rollout.	39	NA	19.31
	No, failure to develop required regulatory documents.	16	NA	7.92

#### 3.3.2 CRC role boundary violations

##### 3.3.2.1 Causes/contributing factors of CRC role violations

Among 202 survey respondents, the primary perceived causes of CRC role boundary violations were (response rate/penetration rate): Investigator’s unclear understanding of role delineation (33.71%/87.62%); Insufficient investigator time commitment to trial implementation (32.38%/84.16%); Low investigator willingness to perform trial responsibilities (18.48%/48.02%). Notably, only 77 respondents (38.12%) attributed role violations to CRC’s own misunderstanding of responsibilities. In addition, when investigating under which circumstances CRCs were more prone to overstepping their positions, the results showed that 181 (89.60%) of the investigators believed that it was the researchers who required CRCs to complete the matters that might lead to overstepping their positions. This study aims to suggest that medical institutions or partners, in addition to providing job responsibility training for CRCs, should also offer relevant training to researchers and clinical trial staff. The training content should be diverse or multi-faceted to enhance their understanding of clinical trials and GCP regulations. The root cause analysis of CRC role violations (summarized in Table 3).

**Table 3:** Causes/contributing factors of CRC role violations.

Group	Variable	*N*	RR (%)	Penetration rate/ percent (%)
Factors contributing to CRC role boundary violations (Valid responses received: 202 participants)	Insufficient investigator time allocation for clinical trial implementation	170	32.38	84.16
	Suboptimal investigator engagement in clinical trial execution	97	18.48	48.02
	Ambiguity in investigator-CRC role delineation	177	33.71	87.62
	Role ambiguity among CRCs	77	14.67	38.12
	Higher contractual compensation for CRCs compared to principal investigators	3	0.57	1.49
	Other	1	0.19	0.5
Total		525	100.00%	259.91%
Contextual scenarios of CRC role expansion behaviors (Valid responses received: 202 participants)	Investigator requests for CRC task overreach	181	38.11	89.60
	CRCs may independently undertake tasks exceeding their predefined scope of duties	88	18.53	43.56
	Supervisory personnel require CRCs to perform tasks potentially beyond their authorized scope of responsibilities	106	22.32	52.48
	CRA requests for CRC task overreach	91	19.16	45.05
	Other	9	1.89	4.46
Total		475	100.00%	235.15%

Penetration rate = (Number of respondents selecting the option / Total valid respondents) × 100%; sum >100% due to multiple choices. Total penetration rate (259.91%) reflects cumulative prevalence of all deviation factors.

##### 3.3.2.2 Major types of CRC role boundary violations

The survey results regarding prevalent CRC role violations are presented in Table 4. Analysis identified five primary violation types, with the following distribution (response rate/penetration rate): the proportions of those who logged into the hospital management system using doctor accounts to write/modify medical records (31.93%/75.25%) and those who randomly studied drugs using doctor accounts (22.90%/53.96%) were the largest. The respondents who wrote reports on serious adverse events and made medical judgments/decisions for intervention researchers accounted for approximately 1/3, and their response rates/penetration rates were 15.76%/37.13 and 14.92%/35.15%, respectively. Fifty-seven (11.97%/28.22%) respondents believed that modifying the original records also frequently occurred in the work of CRCs. Based on the survey results and recent NMPA inspection priorities, one key audit focus is CRC authorization issues, specifically verifying whether CRC role boundary violations exist and identifying their main types. This study has compiled a list of activities that CRCs must never perform in their daily work, as detailed in the Discussion section.

**Table 4:** Major types of CRC role boundary violations.

Group	Variable	*N*	RR (%)	Penetration rate/percent (%)
Primary categories of CRC scope-of-practice deviations (Valid responses received: 202 participants)	Interfere with investigators’ clinical judgment and medical decision-making	71	14.92	35.15
	Modify source documents	57	11.97	28.22
	Log into the EHR system with a physician’s credentials to enter or alter clinical documentation	152	31.93	75.25
	Prepare a SAE report	75	15.76	37.13
	Randomize study drugs using a physician account	109	22.90	53.96
	Other	12	2.52	5.94
Total		476	100.00%	235.65%

Based on the specific circumstances of the survey subjects’ affiliated units, job roles, CRC employment forms, the years of clinical trials undertaken by the units, and the number of clinical trials undertaken each year, a statistical analysis was conducted on the main types of CRC role boundary violations (Detailed breakdown in Table 5). The results demonstrated that across all subgroup analyses, unauthorized use of physician accounts to access hospital management systems for medical record documentation/modification was consistently identified as the most prevalent type of role boundary violation, which aligns with the aforementioned survey findings. All intergroup comparisons were conducted using either *χ*^2^ tests or Fisher’s exact tests (Fisher–Freeman–Halton test). The data revealed no statistically significant differences in pairwise comparisons between groups (*p* > 0.05).

**Table 5:** Statistical analysis of CRC role boundary violations.

Group	Variable	Interfere with investigators’ clinical judgment and medical decision-making	Modify Source documents	Log into the EHR system with a physician’s credentials to enter or alter clinical documentation	Prepare a SAE report	Randomize study drugs using a physician account	*X* ^2^	*p*
Employing organization	Sponsor / CRO	16	11	30	12	13	5.076	0.749
	Clinical trial organization	44	35	101	53	77		
	SMO	11	11	22	12	18		
Years of experience in hosting GCP clinical trials	≤1 year	2	3	3	1	4	8.301	0.942
	1–5 year	14	11	29	20	23		
	6–10 year	12	6	24	11	18		
	10–20 year	6	6	23	10	20		
	>20 year	9	9	21	11	13		
Position	Institutional Director/President	1	0	1	0	1	20.571	0.694
	Director of Clinical Research Operations	27	21	53	24	43		
	Clinical research operations personnel	15	8	34	20	23		
	CRA employed by a Sponsor / CRO	9	7	17	7	8		
	CRC employed by a SMO	10	11	22	12	17		
	Director of Sponsor / CRO	2	2	5	3	4		
	Director of SMO	0	0	2	1	2		
	Healthcare facility-hired CRCs	0	1	6	4	6		
	Investigator(s) at healthcare institutions	5	6	8	4	5		
	Other	2	2	4	1	0		
Average annual volume of GCP-compliant clinical trials conducted	≤5	18	15	34	18	21	15.744	0.993
	6–15	9	7	16	7	11		
	16–30	12	10	25	16	16		
	31–50	11	8	16	10	15		
	51–100	10	7	31	12	28		
	101–150	4	2	11	4	9		
	151–200	5	4	7	3	4		
	201–300	1	2	3	1	1		
	>300	1	2	3	1	1		
Type of appointment	Institutionally-employed CRCs	0	1	6	4	6	4.446	0.169
	SMO-deployed CRCs	10	11	22	12	17		

##### 3.3.2.3 Control measures for CRC overstepping boundaries

The survey questionnaire designed in this study primarily focuses on control measures targeting researchers and CRCs, with their training being mainly provided by medical institutions and SMO companies. Specific survey details are presented in Table 6. The survey results regarding control measures for CRCs and their corresponding percentages (response rate/popularization rate) are as follows: Medical institutions establishing CRC management systems with regular dissemination and assessment (26.85%/79.21%); Medical institutions implementing individual training and assessment systems for externally hired CRCs, requiring qualification before assignment (22.32%/65.84%); Medical institutions creating a blacklist/whitelist system for SMO companies, terminating cooperation with those that frequently fail to manage CRC overstepping (19.46%/57.43%). The survey results regarding control measures for investigators and their corresponding percentages (response rate/popularization rate) are as follows: strengthening the management of investigators to prevent sharing personal accounts with others (29.70%/87.62%).

**Table 6:** Control measures for CRC overstepping boundaries.

Group	Variable	*N*	RR (%)	Penetration rate/ percent (%)
Control measures for CRC scope-of-practice violations (Valid responses received: 202 participants)	Healthcare institutions establish CRC management policies with regular training and competency assessments	160	26.85	79.21
	Healthcare institutions implement individualized training and assessment protocols for external CRCs, with mandatory competency verification prior to task assignment	133	22.32	65.84
	Enhance investigator supervision to prevent credential sharing practices	177	29.70	87.62
	Healthcare institutions establish an SMO accreditation system, terminating collaborations with companies demonstrating persistent CRC protocol violations without corrective	116	19.46	57.43
	Other	10	1.68	4.95
Total		596	100.00%	295.05%
The necessity of regulating CRC scope-of-practice deviations (Valid responses received: 202 participants)	Yes	196	NA	97.03
	No	6	NA	2.97

A total of 196 respondents (97.03%) believed that managing CRC overstepping behavior is necessary. Both medical institutions and clinical trial sponsors should respond immediately upon detecting such behavior to avoid compromising study quality and jeopardizing subject rights and safety. Furthermore, based on the analysis of the above content, strengthening the management and training of investigators accounted for the highest proportion in this survey. Combined with the findings in “Section 3.3.2.1”, CRC overstepping behavior in daily work is mostly passive in nature. When drafting inspection protocols, should regulatory authorities, in addition to specifying CRC responsibilities, also consider the accountability of other relevant parties in their descriptions of overstepping behavior?

#### 3.3.3 Risk and consequence assessment

The survey questionnaire designed in this study categorized the risks and consequences caused by CRC overstepping behavior into three major types: direct risks, impacts on clinical trial institution reputation, and effects on clinical trial quality. Specific details are presented in Table 7. Regarding direct risks (response rate/popularization rate), 186 respondents (36.83%/92.08%) identified increased regulatory compliance risk as one of the primary direct risks, followed by compromised data authenticity (31.09%/77.72%) and infringement of participant rights (22.57%/56.44%). The survey revealed that 42 respondents (8.32%/20.79%) believed CRC overstepping behavior could exacerbate internal conflicts within the research team. Regarding impacts on clinical trial institution reputation, the most significant aspect was the diminished credibility of the institution in the clinical trial field (40.39%/82.18%). Additionally, some respondents believed CRC overstepping behavior could undermine investigators’ medical authority (27.01%/54.95%). The survey of participating medical institutions revealed that the majority have not encountered CRC overstepping behavior to date. However, 15 institutions (7.43%) reported that such violations had already caused irreparable impacts on their clinical trials.

**Table 7:** Risks and consequences of CRC role boundary violations.

Group	Variable	*N*	RR (%)	Penetration rate/percent (%)
Primary risk (Valid responses received: 202 participants)	Impairment of data veracity	157	31.09	77.72
	Violation of participant rights	114	22.57	56.44
	Increased regulatory compliance risk	186	36.83	92.08
	Escalation of intra-team conflicts	42	8.32	20.79
	Other	6	1.19	2.97
Total		505	100.00%	250.00%
Effect on clinical trial site reputation (Valid responses received: 202 participants)	Compromising the investigator’s medical credibility	111	27.01	54.95
	Compromising the trustworthiness of clinical trial sites within the clinical research domain	166	40.39	82.18
	Exerts modest adverse effects	123	29.93	60.89
	Negligible impact	11	2.68	5.45
Total		411	100.00%	203.47%
Effect on clinical trial quality (Valid responses received: 202 participants)	Yes, the problem is inherently irremediable	15	NA	7.43
	Yes, the issue has been rectified	33	NA	16.34
	No	154	NA	76.24

## 4 Discussion

The role of Clinical Research Coordinator has existed for at least two decades both domestically and internationally. However, the status of CRCs and their critical role in clinical trials have only been clearly defined in recent years (12, 22–25). While relevant clinical trial regulations and clinical practice guidelines in China and other countries provide well-defined responsibilities for investigators, sponsors, and clinical monitors, the duties and primary tasks of CRCs have merely reached basic consensus and have yet to be systematically described within a complete framework. A review of the literature reveals relatively limited research publications concerning the CRC profession. The majority of existing papers primarily focus on either discussing the required competencies for CRCs or describing their role-specific responsibilities, particularly in oncology trials (26, 27). Other publications consist mainly of case summaries based on institutional administrators’ experiential knowledge. There remains a paucity of evidence on this topic, with particularly scarce empirical research analyzing CRC overstepping behaviors. Existing literature has demonstrated (28–31) that establishing clear job descriptions and implementing standardized supervision of CRC positions can effectively prevent overstepping behaviors. Such measures contribute to ensuring the authenticity of clinical trial data, enhancing trial quality, and protecting participants’ rights.

Our institution in Chengdu, Sichuan Province has 40 years of experience in conducting clinical trials. As an established research center, we began training in-house CRCs in 2022, who have now accumulated 3 years of work experience. All our in-house CRCs hold bachelor’s degrees in nursing. This is our department’s specific recruitment requirement, not an industry-wide norm. Since their employment, these CRCs have demonstrated significantly stronger regulatory and GCP awareness compared to external CRCs. This study distributed electronic questionnaires, with participation from 30 SMO-employed CRCs and 8 institutionally-employed CRCs. The results revealed that no overstepping behavior occurred among in-house CRCs, while 3 SMO-employed CRCs exhibited protocol violations. Notably, one CRC’s overstepping resulted in irreparable impacts on clinical trial quality. During the initial training phase for in-house CRCs, our institution’s experienced clinical trial professionals provided comprehensive training based on regulatory requirements and institutional SOPs. Additionally, project administrators conducted management-specific training for the CRCs. Each in-house CRC was required to pass competency assessments before being permitted to assume their duties. In the intermediate training phase, when assigned to clinical trial projects, each CRC underwent evaluations of their theoretical knowledge and project-specific understanding through collaboration between the institution and sponsors/CROs. Authorization to assist with projects was only granted after passing these assessments. Initially, project authorization was limited to one project per CRC, with designation as either a lead CRC or assistant CRC. Currently, our in-house CRCs remain in the intermediate training phase. Subsequent training will be implemented based on their actual work performance and developmental needs. This paper recommends that medical institutions with adequate capabilities may consider establishing in-house CRC training programs following our institutional model, with the objectives of: reducing occurrences of protocol deviations in routine practice, enhancing the authenticity of clinical trial data, improving overall clinical trial quality, safeguarding participant rights from infringement. Our finding that formal in-house training reduces CRC deviation aligns with prior research: Cinefra et al. (29) reported a 40% reduction in protocol violations among CRCs who completed standardized training, while Caminiti et al. (30) noted that institutional training improves GCP compliance by 35%. These consistent results underscore the effectiveness of structured training programs for CRC professionalization.

The survey questionnaire designed for this study investigated not only the causes, main types, and control measures of CRC overstepping behaviors, but also proposed the following improvement recommendations based on empirical findings from this investigation (13, 14).

- Regarding systems and supervision: It is recommended to improve industry entry regulations by establishing unified qualification certification and regular training systems. At the administrative supervision level, a CRC practice licensing system should be implemented, requiring step-by-step systematic training before assuming duties. For cases involving serious protocol violations, restrictions should be imposed on continuing similar practice activities.
- Training aspects: Strengthen investigator training programs to enhance GCP regulation awareness and clarify the scope of responsibilities for both investigators and CRCs. Additionally, improve regulatory compliance consciousness and clinical trial proficiency among GCP-certified investigators. Reduce non-GCP related workloads for these investigators to enable greater time allocation and focus on essential GCP trial activities including subject enrollment, trial management, and protocol implementation.
- Sponsor-Institution collaboration: Sponsors should avoid creating pressure for protocol deviations due to project timelines. Institutions should issue warnings to CRCs or study teams with frequent violations. Institutions should establish a credit rating system (red/black list) for CRCs and SMOs - those listed on the blacklist shall be permanently barred from working at the institution. Experienced institutions may develop in-house CRC training programs.
- In terms of personal login credentials: biometric authentication technologies (e.g., fingerprint recognition, facial recognition) should be incorporated into the login process of hospital Electronic Health Records (EHR) and Interactive Web Response Systems (IWRS). Potential challenges include safeguarding the privacy of biometric data and accommodating the operational habits of elderly researchers, so a phased implementation approach is hereby recommended to address these issues.
- Cultural improvement: Introduce clinical trial management courses (e.g., undergraduate education) that incorporate training on various clinical trial roles, thereby shifting the learning phase forward in professional development.

This study conducted a comprehensive analysis of activities that CRCs must absolutely not perform, based on interpretation of industry guidelines and current focus areas of National Medical Products Administration (NMPA) inspections (4, 21, 32–35).

1. At the level of the Center for Food and Drug Inspection of NMPA, key inspection focuses include:
  1. Verification of CRC authorization status.
  2. Identification of any overstepping behaviors.
  3. Documentation of all CRC personnel involved in the trial and their affiliated organizations in inspection reports.
  4. Confirmation that medical records are written by investigators, with particular attention to potential instances of CRCs documenting medical records.
2. For common inspection findings, the key focus areas primarily include:
  1. Randomization systems shall not authorize CRCs to perform operations or use investigator accounts. Subject eligibility assessment must be conducted by investigators and shall not be delegated to CRCs (Special note: CRCs must not be granted access permissions for randomization procedures in systems like IWRS.)
  2. The CRC in the biological sample management process cannot be responsible for sample collection. It can be authorized by PI to carry out sample management-related work such as verification, processing, storage, transfer, transportation, recording of sample storage conditions (such as temperature and humidity), and timely reporting of abnormal situations at each link. While some CRCs hold nursing qualifications, biological sample collection for clinical trials is restricted to licensed healthcare providers [e.g., physicians, registered nurses acting as study nurses (SN)] under China’s GCP (21) and Medical Practitioners Law (36). CRCs (regardless of nursing background) function as study coordinators (SC), responsible for sample management (verification, storage, transportation) but not collection. This distinction is formalized in institutional delegation logs, which require PI authorization for SN-specific tasks.
  3. The distribution of drug management cannot be the responsibility of the CRC. CRC shall not distribute or use any research products at any time. CRC may be authorized and only when authorized in writing to participate in the in-hospital transfer, recovery and return of research products.
  4. CRC cannot fill in the subject screening/enrollment form and the identification code table for the work that needs to be determined by the researcher, such as whether the subjects meet the enrollment conditions and the reasons for dropout. CRC can record the demographic information of the subjects.
  5. CRCs shall never make medical judgments or perform medical interventions. They may address trial process-related questions from participants.
  6. CRC is unable to provide answers to medical-related questions. If it is only an entry error, the CRC should modify the data and answer the doubts. Other medical-related questions should be answered by the researchers.

These findings demonstrate that CRC authorization and responsibility delineation have become critical focus areas, representing frequent non-compliance findings during inspections.

## 5 Limitations

The survey respondents in this study comprised various clinical trial stakeholders, including medical institutions (66.83%), sponsors/CROs (17.82%), and SMOs (15.35%). As the survey was conducted anonymously, the results reasonably reflect these stakeholders’ perspectives on CRC protocol deviations. However, the study was limited to clinical trial practitioners affiliated with hospitals possessing clinical trial institutional qualifications in Sichuan Province. Although the participating sponsors/CROs and SMOs operate nationwide, the sample size remains constrained. Whether these findings represent the broader industry landscape requires further large-scale studies. A notable limitation of the present study is that we did not conduct an analysis of how the number of research projects assigned to each CRC impacts their responsibility deviation. Relevant studies have indicated that (37) there is an upper limit on the number of projects that assigned coordinators can undertake, specifically that each coordinator shall manage no more than 3 projects in the recruitment phase or be responsible for a total of no more than 25 participants in the treatment phase. Yet, we did not investigate whether excessive workload contributes to the occurrence of their responsibility deviation. Future longitudinal studies may help elucidate this causal relationship, as the existing cross-sectional data are unable to determine its directionality. This gap limits our understanding of the organizational factors contributing to such deviation.

## 6 Conclusion

Analysis of the survey questionnaire designed for this study reveals that CRC protocol deviations predominantly correlate with investigators’ insufficient awareness of regulatory requirements and clinical trial standards, coupled with unclear delineation of responsibilities between investigators and CRCs. As investigators, they must unequivocally recognize their essential trial responsibilities, including but not limited to: conducting informed consent processes, making medical judgments, performing medical interventions, documenting medical records. As CRCs, compliance with GCP and industry guidelines is imperative. CRCs must: recognize prohibited activities beyond their scope, refrain from executing unauthorized responsibilities, effectively support investigators in clinical research activities, jointly enhance operational efficiency and trial quality. This dual commitment fosters protocol adherence while optimizing research outcomes.

## Data Availability

The original contributions presented in the study are included in the article/supplementary material, further inquiries can be directed to the corresponding author.

## Footnotes

1
https://www.sojump.com

## Glossary

CRC: Clinical research coordinator
CRA: Clinical research associate
PI: Principal investigator
GCP: Good clinical practice
ICH-GCP: International Council for Harmonisation of Technical Requirements for Pharmaceuticals for Human Use - Good clinical practice
CRO: Contract research organization
SMO: Site management organization
CCA: Complete case analysis
N: Number
RR: Response rate
CVI: Consent validity index
I-CVI: Item-level CVI
S-CVI: Scale-level CVI
S-CVI/UA: Scale-level content validity index / universal agreement
S-CVI/Ave: Scale-level content validity index / average
SAE: Serious adverse event
CRF: Case report form
EHR: Electronic health record
ACRP: Association of clinical research professionals
NMPA: National medical products administration
CFDI: Center Food and drug inspection of NMPA
IWRS: Interactive web response system
SN: Study nurses
SC: Study coordinators
